# Use of derived adipose stem cells to reduce complications of cutaneous scarring in smokers. An experimental model in rats^1^

**DOI:** 10.1590/s0102-865020190060000005

**Published:** 2019-08-19

**Authors:** João Maximiliano Pedron Martins, Fernanda dos Santos de Oliveira, Elizabeth Obino Cirne Lima, Diego Dullius, Isabel Cirne Lima de Oliveira Durli, Everton Hiraiwa, Tulio Serrano, Geciele Rodrigues Teixeira, Paulo Marcos Ribeiro Sampaio, Marcus Vinicius Martins Collares

**Affiliations:** I Fellow Master degree, Postgraduate Program in Surgical Sciences, School of Medicine, Universidade Federal do Rio Grande do Sul (UFRGS), Porto Alegre-RS, Brazil. Design of the study, technical procedures, acquisition and analysis of data, manuscript preparation.; II PhD, Researcher, Laboratory of Embryology and Cell Differentiation Research Center, Hospital de Clínicas de Porto Alegre (HCPA), Brazil. Interpretation and analysis of data, critical revision.; III MD, Plastic Surgery Department, HCPA, Porto Alegre-RS, Brazil. Technical procedures, acquisition of data.; IV PhD, Researcher, Laboratory of Embryology and Cell Differentiation Research Center, HCPA, Porto Alegre-RS, Brazil. Technical procedures, acquisition of data.; V Graduate student, School of Medicine, UFRGS, Porto Alegre-RS, Brazil. Technical procedures, acquisition of data.; VI Researcher, Laboratory of Embryology and Cell Differentiation Research Center, HCPA, Porto Alegre-RS, Brazil. Technical procedures, acquisition of data.; VII Researcher, Laboratory of Embryology and Cell Differentiation Research Center, HCPA, Porto Alegre-RS, Brazil. Interpretation of data.; VIII PhD, Full Professor, Plastic Surgery Department, HCPA, and School of Medicine, UFRS, Porto Alegre-RS, Brazil. Design, intellectual and scientific content of the study; critical revision; final approval the manuscript.

**Keywords:** Stem Cells, Nicotine, Rats

## Abstract

**Purpose:**

To evaluate the use of adipose-derived stem cells (ADSC) in reducing the necrosis area in an experimental model of cutaneous ischemic flap in rats submitted to subcutaneous nicotine injection to simulate a smoker patient.

**Methods:**

In an experimental study, 30 rats were enrolled and divided into two experimental groups of 15 animals all submitted to a subcutaneous nicotine injection to create ischemic cutaneous flaps on their backs. Other 10 animals were used only to obtain adipose tissue derived stem cells (ADSC). The first group (n=15) received ADSC treatment at the end of surgery while the other group, the control (n=15), received no other interventions. After euthanasia, a decal was performed on the whole area of the flap, accurately defining the transition from necrosis to healthy region. Photos of all animals were collected and evaluated by scales standardized by Paint-Autocad- 2015 software to define the area of flap necrosis in each rat. Student T test was performed to compare the groups, considering a p< 0.05 significant. Data were analyzed using SPSS IBM^®^ 18 version.

**Results:**

Through the analysis of the images by the program Paint-Autocad-2015 and the area of decal obtained by the transparent sheet, we obtained a mean of 46% necrosis of the total area of the flap in the treatment group and 69.4% in the control group. In the descriptive analysis, a mean of 3.7 cm of necrosis CI 95% (3.2 - 4.2) was evident in the treatment group whereas a mean value of 5.56 CI 95% (5.2 - 5.9) was found in control group, with p value <0.001 for this comparison.

**Conclusion:**

The application of adipose-derived stem cells reduces the percentage of necrosis in an experimental model of randomized cutaneous flap in rats submitted to subcutaneous nicotine injection.

## Introduction

Cutaneous scarring is a complex phenomenon, in which small changes can lead to an undesirable pathological outcome. Intercurrences in this process are frequent in plastic surgeries, sometimes leading to unsightly results and frequently requiring reinterventions. A common and well-described complication in the literature is cutaneous necrosis secondary to chronic smoking and patients’ difficulty in stopping it before surgery. About 5% of patients are able to stop smoking before surgery, and this number increases to 15% after medical advice^1^. If active in the pre and postoperative periods, smoking generates an environment of ischemia and hypoxia of the surgical wound. Mainly because nicotine, one of the components of tobacco, which generates vasoconstriction in the microcirculation of the skin, decreases the distal blood flow in the flaps, which results in cutaneous necrosis and subsequent secondary infection^2^. Maintaining smoking in an elective surgical procedure can lead to a risk of delayed healing, dehiscence and infection up to three times higher in comparison to nonsmokers, which often leads to partial or total loss of flaps, secondary scarring and a negative impact on the aesthetic outcome^3,4^. Several clinical studies and experimental models associating tissue necrosis of flaps with smoking have been described^5-8^. To allow the evaluation of the damages caused by smoking, experimental models that induce the complications caused by smoking were created, in cutaneous flaps, through the subcutaneous injection of nicotine, without the need to produce toxic smoke, facilitating the realization of the studies^9^. The application of the Adipose Derived Stem Cells (ADSCs) in the cutaneous wound improves cicatrization by cell differentiation and mainly by its important improvement of paracrine action, increasing the stimulation to neovascularization and regulating the coagulation cascade^10^. The objective of the study was to test the use of ADSCs in an experimental model of a randomized cutaneous flap in rats with subcutaneous nicotine injection to reduce necrosis area.

## Methods

The research ethics committee of Hospital de Clínicas de Porto Alegre - UEA - UFRGS, analyzed and approved the research protocol No. 2016/0460 related to this experiment.

This is an experimental study carried out at the Laboratory of Embryology and Cell Differentiation, at the Animal Experimentation Unit (UEA) and at the Experimental Pathology Unit, located at the Experimental Research Center of the Hospital.

Forty male rats, *Rattus norvegicus albinus*, *Rodentia mammalia*, Wistar lineage, weighing 250 to 300g young adults, at least 8 weeks, were obtained according to the criteria of the HCPA Animal Experimentation Unit. The rats were housed in the UEA-HCPA, according to the use pattern of this unit, where they received commercial ration and water *ad libitum*, under controlled temperature (22ºC ± 2°C) and air relative humidity (55% ± 5%) and photoperiod of 12 / 12 hours. Prior to initiating procedures, the animals were quarantined and acclimated for at least 15 days. In vivo procedures were carried out in accordance with Brazilian legislation, Law 11.794 (Official Gazette of the Union - 8/10/2008), which establishes procedures for the scientific use of animals and regulates the registration of Animal Houses and Experimental Centers. All procedures were based on the Brazilian Guideline for the Care and Use of Animals for Scientific and Educational Purposes - DBCA (2016). The completion procedures follow the standards indicated by the CONCEA Guidelines for Euthanasia Practice (2013).

### Experimental groups

Thirty rats were divided into two experimental groups of ischemic cutaneous flap on their back injected with subcutaneous nicotine. The first group (n = 15) received ADSCs treatment at the end of surgery and the other group was the control group that received only solution saline (n = 15). Both groups underwent euthanasia 7 days after surgery.

### Nicotine injection

The animals treated with nicotine (Nicotine hemisulfate salt (−)-1-Methyl-2-(3-pyridyl)pyrrolidine SIGMA-ALDRICH, Brazil) were injected into their subcutaneous tissue at a dose of 1.2 mg / kg / day, which corresponds to a dose equivalent to a heavy human smoker for a period of 7 days before surgery after the intervention, until the day of euthanasia without interruption11.

### Collection and processing of adipose tissue to obtain mesenchymal stem cells

For the collection of adipose tissue, 10 rats, *Rattus norvegicus albinus*, Rodentia, Wistar lineage Mammalia, weighing 250-300 g, young adults, 4-8 weeks young, were obtained and maintained as previously described. The animals had gonadal and abdominal adipose tissue collected aseptically, under general inhalation anesthesia, with Isoflurane, vaporized in 100% oxygen, at a dose of 5% for induction and 2% for maintenance, at 0.5L / min. After collection, euthanasia was performed through anesthetic overdose with isoflurane until the promotion of cardiorespiratory arrest. The fragments of adipose tissue were immediately sent to the Laboratory of Embryology and Cell Differentiation at the Experimental Research Center of HCPA, where they were submitted to enzymatic digestion for the isolation of ADSCs.

### Obtaining MSC

In a laminar flow hood, tissue from the donor animals was incubated in collagenase type I solution (1mg.mL-1 in DMEM 9mM HEPES) for a period of one hour at 37°C. After, the enzyme was inactivated by the addition of Dulbecco’s modified Eagle’s medium (DMEM) culture medium supplemented with 10% fetal bovine serum (FBS). The cell suspension was centrifuged for 5 min at 2000 rpm, and the formed pellet was suspended again in DMEM medium, supplemented and plated. After isolation, the cells were cultured in DMEM, containing a low glucose concentration, supplemented with 15mM HEPES 9mM, 15% FBS and antibiotic solution of 100 units.mL-1 of penicillin and 100mg.mL-1 of streptomycin, at 37°C, in an atmosphere of 5% CO2 and 100% humidity. After 24 hours of cultivation, the culture medium was aspirated and fresh medium added. When the cell culture reached 80% confluence, the adherent cells were removed with 0.05% trypsin-EDTA solution and subcultured in DMEM. The stem cells presented the properties for the characterization as ADSCs. In the flow cytometry immunophenotyping assay, monoclonal antibodies against rat antigens, demonstrated that cultured cells presented a 99% expression of CD90, CD73, and CD29, in addition to minimal reactivity (<2%) to negative markers (CD45 and CD11b). In vitro-induced differentiation assays were performed, using 21 days and subsequent specific staining of cells; stem cells were able to differentiate into adipocytes, chondrocytes, and osteocytes. The cells used were between the 3rd and 5th passage.

### Surgical model

**Figure 1:**
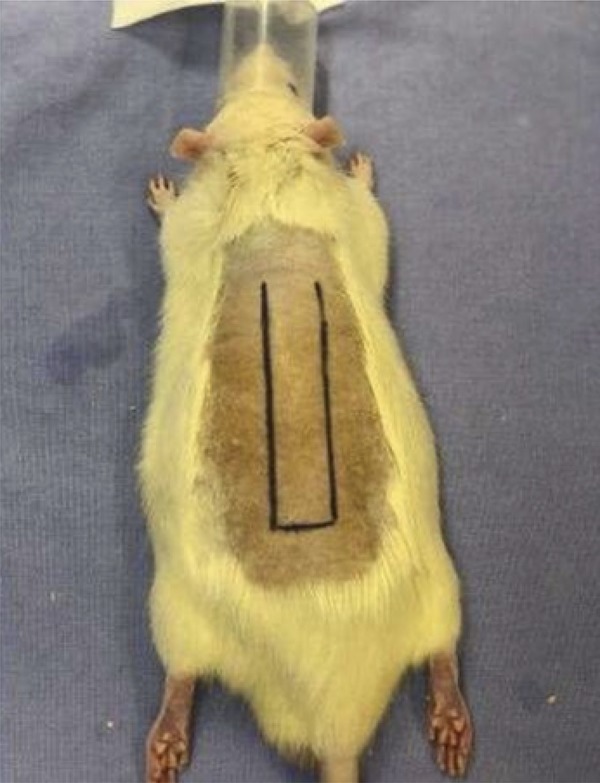
Detached cutaneous retail template (8x2cm).

**Figure 2:**
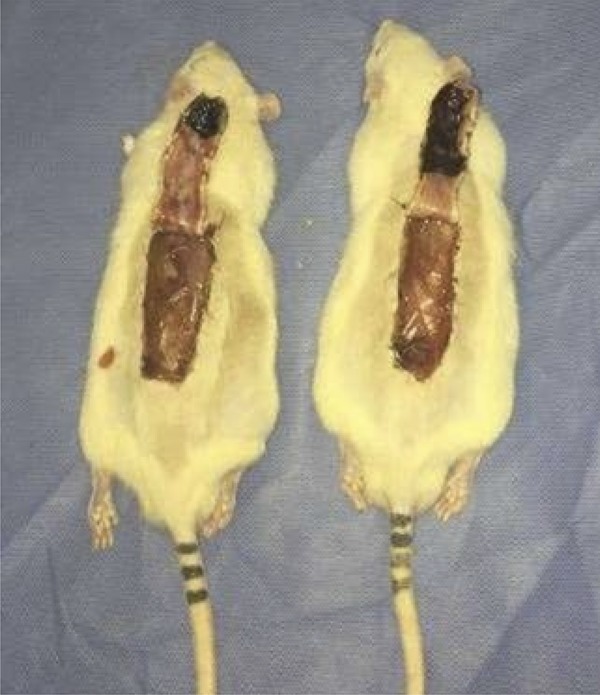
Plastic barrier properly positioned at the time of euthanasia. Rat left group treatment, right mouse control group.

The animals were submitted to general inhalation anesthesia with Isoflurane, vaporized in 100% oxygen, at a dose of 5% for induction and 2% for maintenance, at 0.5 L/min. For analgesia, tramadol hydrochloride (20mg.kg-1) was administered intraperitoneally (IP) and after induction of lesions, every 12 hours until euthanasia. A wide trichotomy of the dorsal region was performed, and the animals were placed in a ventral decubitus position to perform antisepsis of the surgical field with alcohol solution of 2% chlorhexidine gluconate. An experimental model already described in the literature, cranial base flap, 8cm long and 2cm wide, was used on the back of the animals. The flap is bounded by a line that joins the lower angles of the shoulder blades and the upper edges of the pelvic girdle bones. The flaps were raised from the superficial fascia of the skeletal muscles containing the skin and subcutaneous tissue (Fig. 1). A plastic barrier (polyester/polyethylene) with the same dimensions (8x2) was interposed between the flap and the donor bed, preventing revascularization of the flap by means of bedding vessels (Fig. 2).

Next, the flap was sutured with single stitches of 4-0 monofilament nylon thread in the same original position (“donor area”), spaced 1cm apart between stitches^9,11-15^. In the ADSCs treatment group, cells were used in the approximate concentration of 1X106 cells, suspended in 1ml of 0.9% NaCl. After finishing the suture, it was injected intradermally throughout the flap, with 1ml syringe and 27gauge needle, being 0.1ml every 1.5cm on the side of the flap, totalizing 0.4ml on each side of the flap and 0.2ml in the distal portion of the flap. In the control group the same procedure was performed, however only with saline solution. After the procedure the animals were observed every 8 hours for the first 48 hours and thereafter daily for the remainder of the 7 day experiment. The mouse patterns were noted after the procedure to report any change in appetite or behavior. Death-anticipation measures were defined as a refinement procedure and protection/preservation of animal welfare, if the animals presented altered behavior, such as excessive weight loss, anorexia, signs of pain that could not be treated, following the recommendations of CONCEA (2013). There was no loss of the animal in the postoperative period and it was not necessary to anticipate euthanasia due to failure of the analgesia protocol.

### Euthanasia

The animals were submitted to euthanasia with anesthetic overdose with Isoflurane, at a dose five times higher than the therapeutic dose, until the promotion of cardiorespiratory arrest, confirmed by a trained Veterinarian.

### Necrosis area evaluation

**Figure 3:**
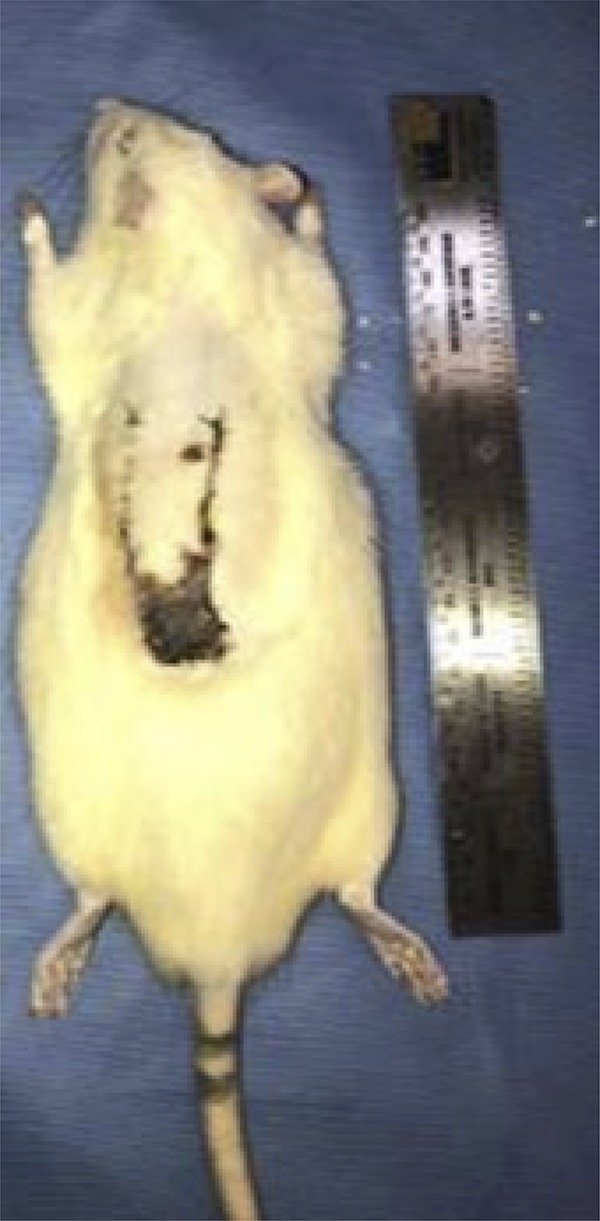
Mouse of the control group at the time of euthanasia.

**Figure 4:**
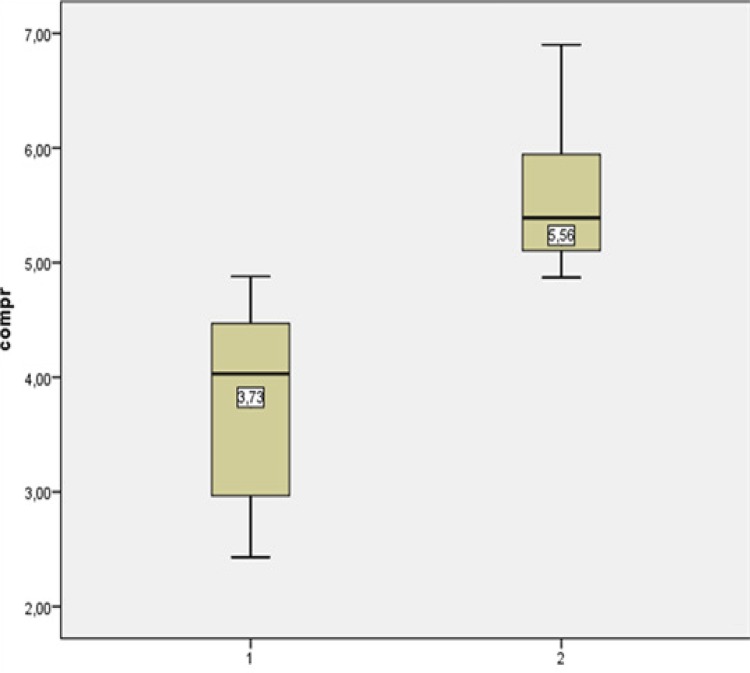
Rat of the treatment group at the time of euthanasia.

After euthanasia, a decal of the whole area of the flap was performed, accurately defining the transition from necrosis to the healthy region. For this, a transparent plastic sheet was placed on the back of the animal and with pen-marking the area of necrosis was marked and measured. By dividing the area value obtained for necrosis by the total flap area and multiplying the result by 100, the percentage of necrosis of the flap total area is obtained. In addition, all animals were photographed and evaluated by Scales standardized by the Paint-Autocad-2015 program and performed patchy patch analysis, recording in square centimeters the total area, area of viable vascularized tissue and area of necrotic tissue (Figs. 3 and 4)^16^.

### Statistical analysis

For statistical analysis, IBM SPSS version 18 software (SPSS Inc. IBM Company) was used. The main outcome variable was the area of necrosis, measured in cm2. For continuous and normal distribution variables, we used the Student’s T test. The results were expressed as mean and standard deviation. We considered a significant difference when p <0.05 and the 95% confidence interval did not cross 1.00.

## Results

### Comparison of the area of necrosis of the flap

There was no case that required measures of anticipation of death due to failure of the analgesia protocol, following CONCEA recommendations (2013). There was no loss of animal in the postoperative period. Two animals had episode of epistaxis after injection of nicotine and one convulsed, without need of any intervention.

Through the analysis of the images by the decal area obtained by the transparent sheet and the Paint-Autocad-2015 program, the necrosis area of the flap ranged from 30% to 61% in the treatment group, whereas in the control group it varied from 61% to 86%. An average of 46% necrosis of the total flap area was obtained in the treatment group and 69.4% in the control group (Table 1).

**Table 1:** Results of the necrosis area in the rats of the treated group and control group.

Treated group (n=15)	Necrosis area (%)	Control group (n=15)	Necrosis area (%)
1	35	1	61
2	56	2	64
3	50	3	74
4	33	4	61
5	59	5	81
6	56	6	64
7	56	7	66
8	42	8	69
9	58	9	66
10	34	10	71
11	50	11	62
12	61	12	74
13	30	13	76
14	40	14	86
15	39	15	67

In the descriptive analysis, a mean of 3.7 cm of necrosis (CI 3.2 - 4.2) and a mean of 5.56 (CI 5.2 - 5, 9) p <0.001 (Table 2).

**Table 2:** Results of mean necrosis length in treated and control groups.

Variable	Treatment *µ* (DP)	Control *µ* (DP)	*p*
Length of necrosis (cm)	3.7 (±0.86)*	5.56 (±0.61)*	0.001**

* Data with normal distribution represented by mean and DP.

** Data obtained from the comparison of the means of the groups through Student’s T-Test.

**Figure 5:**
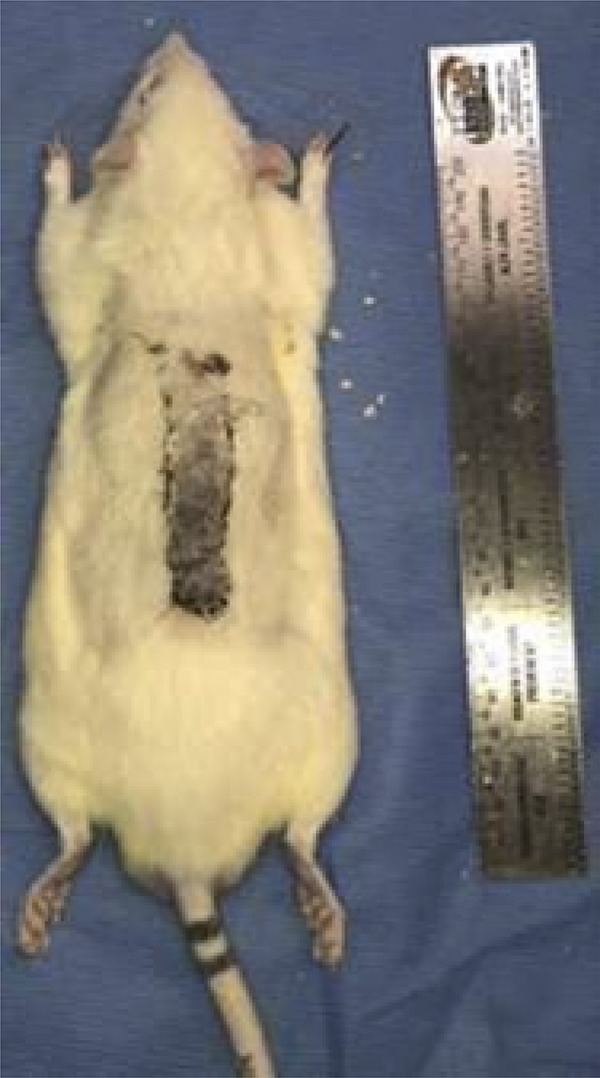
- Mean and standard deviation in cm in the treatment group (1) and control group (2).

The mean and standard deviation of the necrosis area in the treatment and control group are plotted on the Boxplot graph which was used to evaluate the distribution of the data (Fig. 5).

## Discussion

Cigarette smoke is made up of nearly 4.000 chemicals, most of which are toxic to the body. When the skin flap vascularization is studied in smokers, the main harmful substance is nicotine because it reduces perfusion of the flaps by vasoconstriction^17^, increased platelet adhesiveness^18^ and reduced coagulation time^19^. The cutaneous flap is affected by intrinsic and extrinsic factors. Extrinsic factors can be systemic (infection, arteriosclerosis, hypotension and nutritional deficiency) or local (compression, tension or thrombosis of the anastomosis). The main intrinsic factor is inadequate blood flow due to arterial insufficiency in the distal part of the flap. Nicotine treatment in experimental skin flap models results in vasoconstriction, reducing blood flow, especially to the distal flap. This drug also impairs the healing of wounds in the inflammatory and epithelial phases^11^. Several measures have been described in an attempt to minimize the deleterious effects of smoking on the vascularization of the flaps, due to their potential for damage and mainly because even after counseling, 85% of patients continue to smoke before and after surgery^1^. The authors use the experimental model of mouse cutaneous flaps well established in the literature as a basis for testing new treatments^12^, adding nicotine injection in the subcutaneous to simulate a chronic smoker individual.

Russo *et al*.^11^ described the use of transcutaneous electrical nerve stimulation (NTG) demonstrating increased viability of the cutaneous flap in rats treated with nicotine, 45% of mean necrosis in the control group and 21% in the group exposed to NTG, due to improvement of blood flow. Leite *et al*.^20^ used dimethyl sulfoxide as a blocker to the deleterious effect of nicotine demonstrating a mean area of necrosis of 20% while in the control group 40%, concluding that due to the specific antioxidant effect, the drug blocked the harmful effect of nicotine, acting primarily on the hydroxyl radicals, considerably reducing the deleterious effect of nicotine. Uzun *et al.* ^21^ tested the use of Vareneclin before raising the flaps, describing a significant improvement in vascularization and percentage of flap necrosis, 49% in the control group and 22% in the Vareneclin group. Also, microangiographically showed that the vascularization was lower in the nicotine group and higher in the treatment group. Histologically, larger areas of necrosis, more severe inflammation and less vessel formation were observed in the nicotine group. Guimarães *et al.* ^16^ described that with arginine treatment at 300 mg/kg in rats exposed to nicotine, vessels counted through immunohistochemistry with anti-CD31 had a lower mean (2.9), when compared to the treatment group (4.3). The mean area of necrosis in the treated group was 17% while the mean of the control group was 45%. They attributed the effects of Arginine to a consequence of the drop in the blood concentrations of pyruvate, lactate and ketone bodies. Shah *et al*.^22^ tested the ability of increased vasculogenesis and increased blood flow generated by the inhibition of phosphodiesterase-5 (PDE-5) in reducing the complications generated by nicotine. It concluded that there was a statistically significant reduction in the area of necrosis of the skin flap in rats exposed to nicotine when PDE-5 inhibited; this effect was dose dependent. When administered sildenafil twice daily, the animals exposed to nicotine had a reduction of more than 50% in cutaneous necrosis when compared to the control group, in a statistically significant way. When VEGF was analyzed, the amounts produced in the treatment group, mean of 4.0, were also statistically higher than in the control group, 2.9 of mean. Rinker *et al*.^23^ tested the effect of calcium channel blockers on the percentage of necrosis area of the tobacco-induced skin flap in rats, and concluded that calcium channel blockers, both verapamil and nifedipine, administered at the time of surgery and post- of the skin flap necrosis in animals exposed to nicotine, produced 27.2% and 26.3%, respectively, when compared to the control 36.3% of the mean. Selçuk *et al*.^24^ used the therapy with hyperbaric oxygenation to reduce the area of necrosis in cutaneous flaps of rats exposed to nicotine, describing reduction with statistical relevance in the treatment group of 35.9% and in the control group 53.9%. Nezami *et al*.^25^ demonstrated that single, local and low doses of tacrolimus reduced the area of skin flap necrosis in nicotine treated rats significantly, 28.3% in the treatment group and 53.9% in the control group by reducing the oxidative stress produced by nicotine.

This study aims to test the use of ADSCs as a prophylaxis of vascular complications caused by nicotine in ischemic cutaneous flaps in rats. The difficulty of patients in stopping smoking follows a recurring problem in the routine of plastic surgeons. It is already proven that most of the patients continue smoking even after preoperative orientation, as well as the various complications that smoke generates in the vascularization of the tissues, cutaneous scarring, which can lead to serious complications and need for reoperation^1-4^. The application of the stem cells to the wound generates an important improvement of the cicatricial paracrine action, significantly increasing the secretion of VEGF and Ang1 in wounds up to five times. The elevation of these proangiogenic factors leads to the improvement of neovascularization^26^. Thus, in flaps with ischemic potential, such as that of smokers, they can be used as a therapeutic option to increase neoangiogenesis and reduce necrosis secondary to vasoconstriction generated by nicotine.

For stem cell treatment, cells derived from adipose tissue were chosen. They are easily accessible in the routine of plastic surgeons and lower morbidity when compared to bone marrow. In addition, they can yield 500 times more cells from the same amount of tissue andare genetically more stable in long-term cultures^27^; and because of the large amount of tissue available, liposuction can be directly used clinically without culture in vitro^28^. The direct use of liposuction can be safer, since it avoids the in vitro manipulation of ADSCs that could alter their biological functions. Another advantage of using liposuction without passing through culture are the extremely rigid regulatory issues of stem cell management and subsequent clinical application, which require high cost and a properly trained staff^29^. Also, they can be cryopreserved for up to 6 months while maintaining their characteristics and allowing their therapeutic use in the future^30^. Therefore, its benefit in clinical application would be more feasible when compared to the other sources of stem cells.

This experimental model was chosen after a pilot project where 10x4cm and 8x2cm flaps were tested and the nicotine concentrations of 2 mg/kg/day and 1.2 mg/kg/day were tested. As it was not possible to handle the analgesia in the group with 10x4cm flaps and as there were frequent seizures in the 2 mg/kg/day group of nicotine, it was chosen to use the 8x2cm experimental model and the nicotine dose of 1.2 mg/kg/day, which simulates the concentrations of a heavy smoker^9,11-15^.

We found a larger mean area of 8x2cm patch necrosis performed in this study when compared to the other studies described. In a random skin flap in 10 x 4cm rats with subcutaneous nicotine injection (2.5: 1 length / base ratio) a mean necrosis of 40% of the total cutaneous flap area is expected^11^. However, in the flaps with 8cm of length by 2cm of base the proportion length / base is greater 4: 1, then a higher percentage of necrosis is expected; in the literature they are described in average 40% without addition of nicotine^13-15^. A description of an experimental model of a rare cutaneous flap in 8x2cm rats with nicotine addition was not found in the literature, which explains the percentage of necrosis in the total area of this study being higher than the others reported in the literature, 69.4%.

With ADSCs treatment we significantly reduced the necrosis area of the flap from 69.4% in the control group to 46% in the treatment group (p<0.05). There was an average of 23.4% reduction of the necrotic area. Although a significant reduction in the proportion of necrosis area reduction was obtained, it was lower than the other studies analyzed. In this study, there was a reduction of 69% to 46% of the total area of the flap, representing a reduction of 34% of the area of necrosis in the treatment group, while the majority of the other studies obtained a reduction of necrosis from 40% to 20% of the total area of the flap, and a 50% reduction of the area of necrosis in the treatment group. However, we believe that more studies are needed using the 8x2cm model with nicotine, since the rats adequately tolerated a greater area of necrosis of the flap; then, there is a greater margin for testing new treatments and their potential for vascularization improvement. There are limitations in this study; although nicotine is the major byproduct of cigarette smoke, nicotine administration in the subcutaneous may not represent the actual conditions of a smoker. Alternatively, passive smoking chambers could be used to ensure the presence of other constituents of cigarette smoke; however, the use of the chamber was not approved by the hospital’s ethics committee because of the risk of toxic smoke contaminating other non-involved animals and professionals who participated in the research. In addition, the exposure frequency proposed in experimental models with passive smoke does not correspond to that of smokers, and confinement of animals in a chamber may result in hypoxia.

Because fat liposuction is a recurring practice in the plastic surgeon’s routine and has low morbidity, the application of ADSCs, without isolation and proliferation in vitro pre-treatment, can be routinely aggregated as prophylaxis of complications in cutaneous flaps, still requiring studies to verify the findings found in this experimental model.

## Conclusion

The use of ADSCs in an experimental model of a randomized cutaneous flap in rats with subcutaneous nicotine injection reduces the percentage of necrosis of the flaps.

## Footnotes

1 Research performed at the Laboratory of Embryology and Cell Differentiation, Animal Experimentation Unit (UEA), and Experimental Pathology Unit, Experimental Research Center, Hospital de Clínicas de Porto Alegre (HCPA), School of Medicine, Universidade Federal do Rio Grande do Sul (UFRGS), Porto Alegre-RS, Brazil. Part of Master’s degree thesis, Postgraduate Program in Surgical Sciences. Tutor: Marcus Vinicius Martins Collares.
